# Supplementary material for: Requirement for YAP1 signaling in myxoid liposarcoma
Source: EMBO Mol Med. 2019 Mar 21;11(5):e9889. doi: 10.15252/emmm.201809889 (PMC6505681; doi:10.15252/emmm.201809889)
Supplement: Supplementary file 1 — Appendix [file EMMM-11-e9889-s001.pdf]

Requirement for YAP1 signaling in myxoid liposarcoma

Marcel Trautmann, Ya-Yun Cheng, Patrizia Jensen, Ninel Azoitei, Ines Brunner, Jennifer Hüllein, Mikolaj Slabicki, Ilka Isfort, Magdalene Cyra, Ruth Berthold, Eva Wardelmann, Sebastian Huss, Bianca Altvater, Claudia Rossig, Susanne Hafner, Thomas Simmet, Anders Ståhlberg, Pierre Åman, Thorsten Zenz, Undine Lange, Thomas Kindler, Claudia Scholl, Wolfgang Hartmann & Stefan Fröhling

Correspondence

- Wolfgang Hartmann, Division of Translational Pathology, Gerhard-Domagk-Institute of Pathology, Albert-Schweitzer-Campus 1, D17, 48149 Münster, Germany
  Phone: +49-251-83-58479; Fax: +49-251-83-55481
  E-mail: wolfgang.hartmann@ukmuenster.de
- Stefan Fröhling, NCT Heidelberg, Im Neuenheimer Feld 460, 69120 Heidelberg, Germany
  Phone: +49-6221-56-35212; Fax: +49-6221-56-5389
  E-mail: stefan.froehling@nct-heidelberg.de

Running title: YAP1 signaling in myxoid liposarcoma

Appendix - Table of contents

- Appendix Figure S1
- Appendix Figure S2
- Appendix Figure S3
- Appendix Figure S4

- Appendix Table S1
- Appendix Table S2

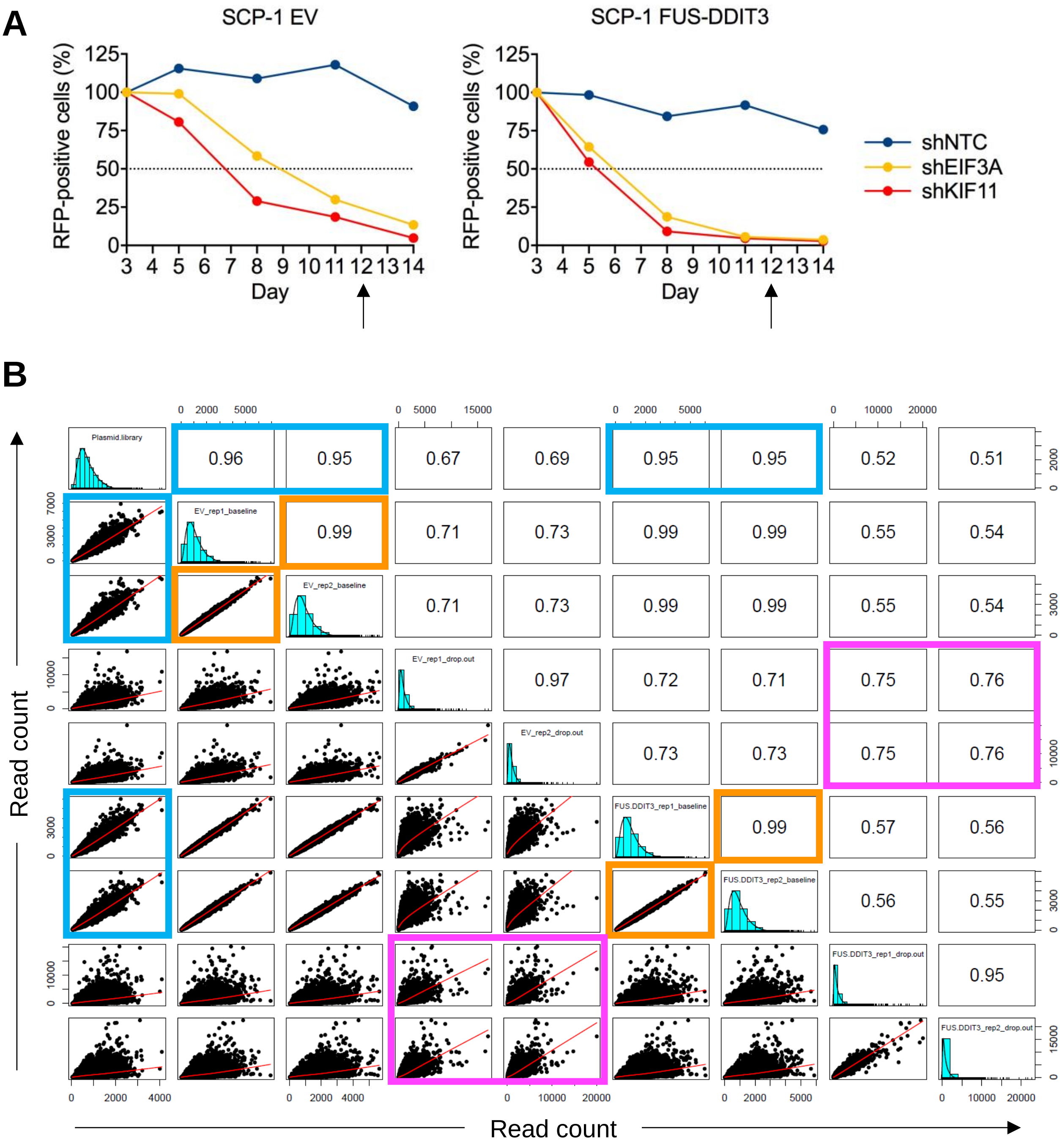

Appendix Figure S1

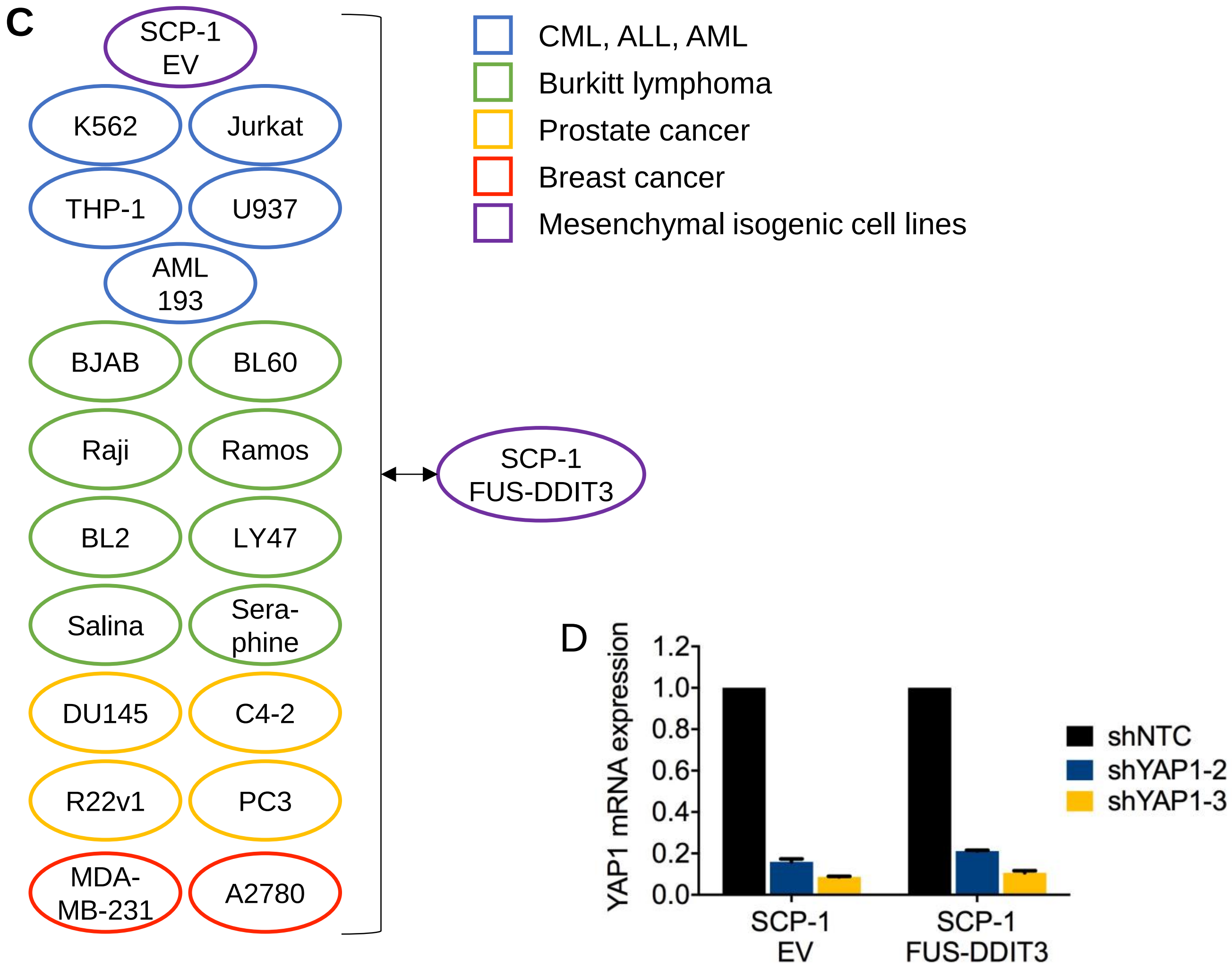

Appendix Figure S1 (related to Figure 1). (A) RFP competition assays with SCP-1 cells transduced with a NTC shRNA or shRNAs targeting the essential genes *EIF3A* and *KIF11* for determining the time point for shRNA screening. Flow cytometric quantification of RFP-positive cells relative to day 3 at different time points showed efficient shRNA-mediated depletion of both SCP-1 cell lines after 12 days (arrows). (B) Correlation of read counts from all shRNA screening samples. Left panels show correlation plots in which each dot represents an individual shRNA. Right panels indicate the corresponding Pearson correlation coefficients. Middle panels show the histogram plots of the read counts from each sample. Blue, correlation between plasmid library and baseline samples; orange, correlation between biological replicates from the baseline samples; purple, correlation between the EV drop-out samples and the FUS-DDIT3 drop-out samples. Rep: replicate. (C) Cell lines used for shRNA screening to identify FUS-DDIT3-specific essential genes. (D) *YAP1* mRNA expression in isogenic SCP-1 cells five days after transduction with YAP1 shRNAs used for the experiment shown in Figure 1D. *YAP1* expression was normalized to *ACTB*.

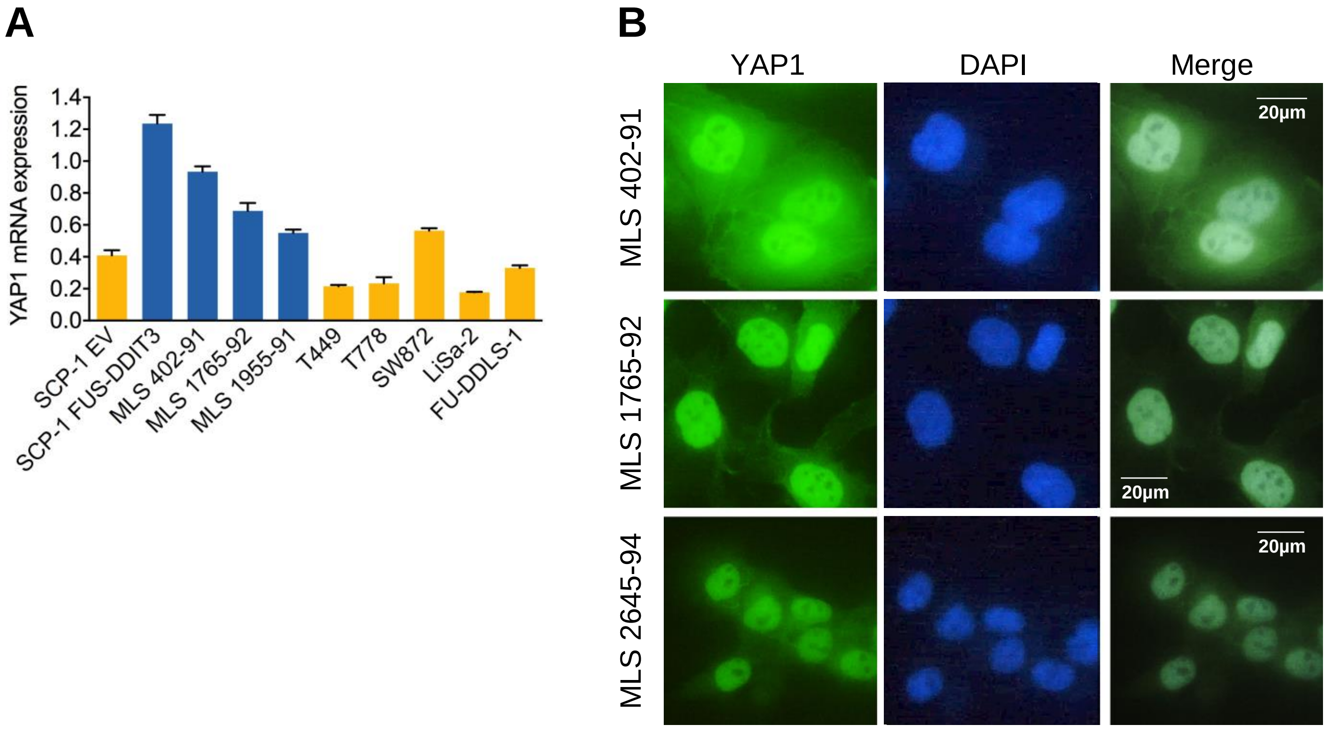

Appendix Figure S2 (related to Figure 2). (A) *YAP1* mRNA expression relative to *ACTB* in isogenic SCP-1 cells and liposarcoma cell lines. Cells expressing FUS-DDIT3 are indicated in blue. (B) IF staining of YAP1 (green) and nuclei (blue) in MLS cell lines.

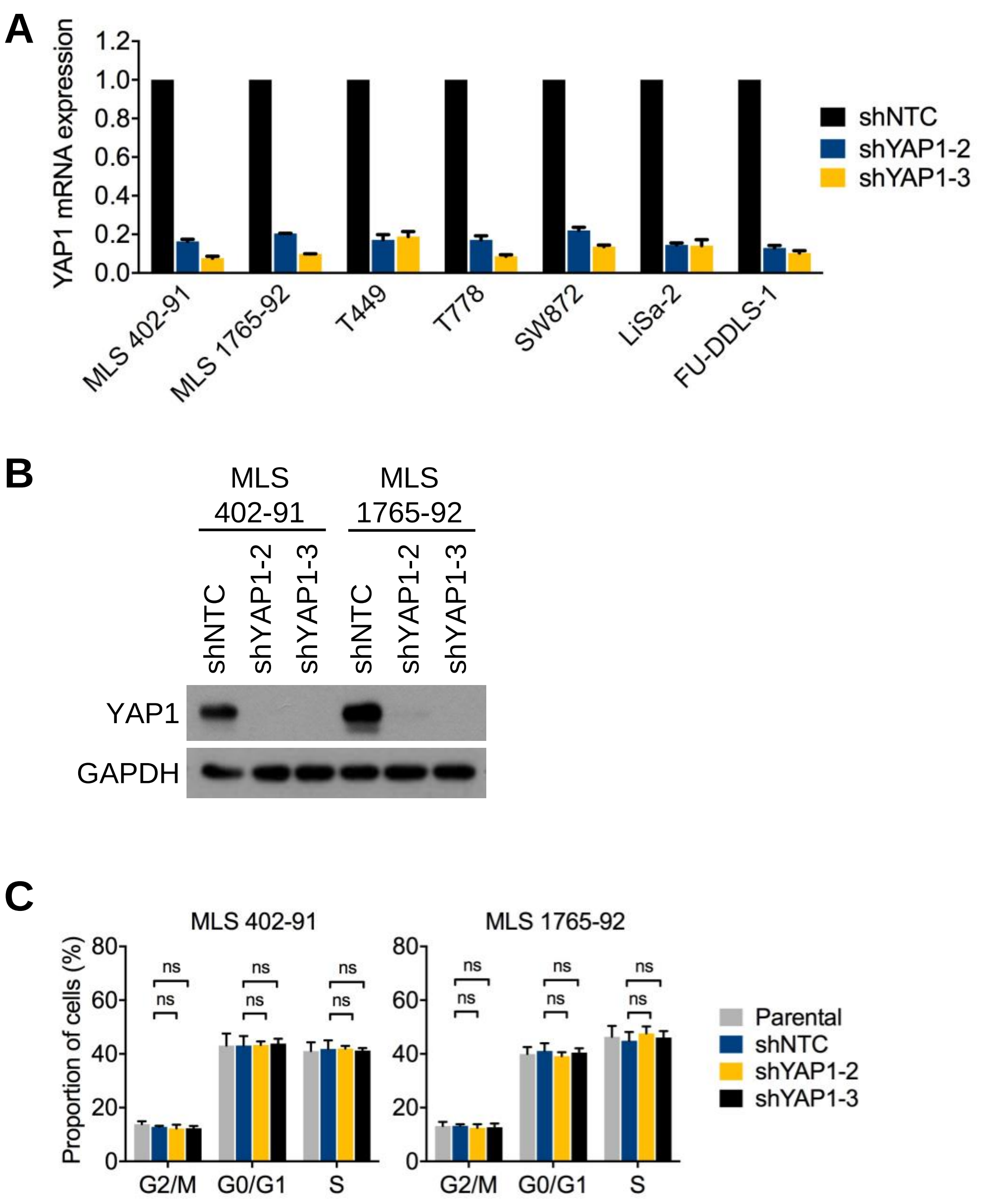

Appendix Figure S3 (related to Figure 3). (A) *YAP1* mRNA expression in liposarcoma cell lines after transduction with *YAP1* shRNAs used for the experiment shown in Figure 3A. *YAP1* expression was normalized to *ACTB*. (B) YAP1 protein expression in two representative MLS cell lines after transduction with *YAP1* shRNAs. (C) Flow cytometric cell cycle analysis of MLS cell lines shown in Figure 3F, gated on RFP-negative untransduced cell populations. Error bars represent the mean ± SD of three independent experiments. ns, not significant.

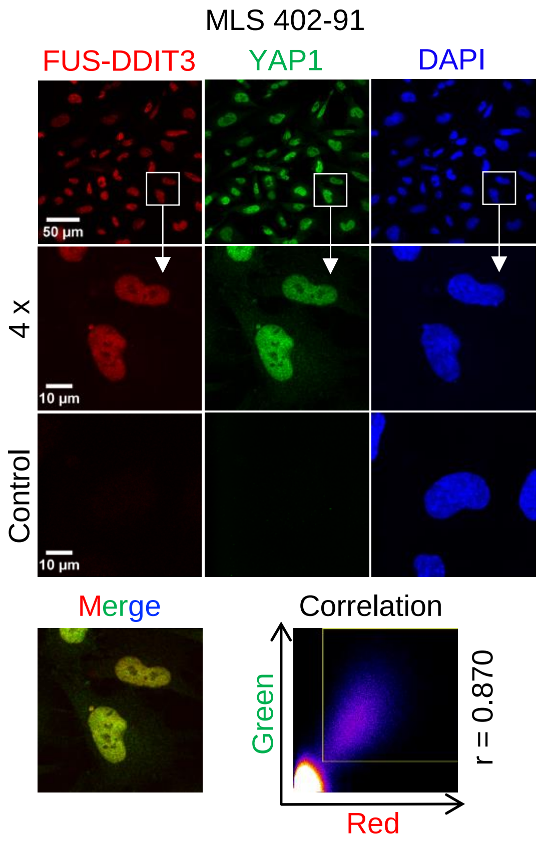

Appendix Figure S4 (related to Figure 4). Localization of FUS-DDIT3 and YAP1 in MLS 402-91 cells. Nuclei were counterstained with DAPI. The original magnification was x63, and images were zoomed in four times for co-localization analysis. The correlation between red and green fluorescence was determined by Pearson coefficient analysis (square, area for signal acquisition).

Appendix Table S1. Primers used for quantitative RT-PCR

Gene	Forward primer	Reverse primer
ACTB	CTCTTCCAGCCTTCCTTCCT	AGCACTGTGTTGGCGTACAG
YAP1	GTTGGGAGATGGCAAAGACA	ACGTTCATCTGGGACAGCAT

Appendix Table S2. Antibodies used for immunoblotting

Antibody	Manufacturer	Order number	Dilution
β-actin	Sigma-Aldrich	A5441	1:1000 in 5% BSA/TBST
β-tubulin	Cell Signaling	2146	1:1000 in 5% BSA/TBST
CDKN1A	Cell Signaling	2947	1:1000 in 5% BSA/TBST
CDKN2A	Abcam	ab108349	1:1000 in 5% BSA/TBST
Cleaved caspase 3	Cell Signaling	9662	1:1000 in 5% BSA/TBST
Cleaved caspase 8	Enzo	ALX-804-242	1:1000 in 5% BSA/TBST
Cleaved PARP	Cell Signaling	9541	1:1000 in 5% BSA/TBST
DDIT3	Cell Signaling	2895	1:1000 in 5% BSA/TBST
FOXM1	Cell Signaling	500	1:1000 in 5% BSA/TBST
GAPDH	Santa Cruz	sc-25778	1:1000 in 5% BSA/TBST
Histone H3	Cell Signaling	4499	1:1000 in 5% BSA/TBST
Lamin A/C	Cell Signaling	2032	1:1000 in 5% BSA/TBST
Pan-TEAD	Abcam	ab197589	1:1000 in 5% BSA/TBST
PLK1	Cell Signaling	4513	1:1000 in 5% BSA/TBST
RB1	Cell Signaling	9309	1:1000 in 5% BSA/TBST
Phosphorylated RB1 (S807/811)	Cell Signaling	9308	1:1000 in 5% BSA/TBST
TP53	Cell Signaling	2527	1:1000 in 5% BSA/TBST
V5	Invitrogen	R960-25	1:1000 in 5% BSA/TBST
YAP1	Cell Signaling	14074	1:1000 in 5% BSA/TBST
